# The carboxy‐terminus of the human ARPKD protein fibrocystin can control STAT3 signalling by regulating SRC‐activation

**DOI:** 10.1111/jcmm.16014

**Published:** 2020-10-28

**Authors:** Claudia Dafinger, Amrei M. Mandel, Alina Braun, Heike Göbel, Kathrin Burgmaier, Laura Massella, Antonio Mastrangelo, Jörg Dötsch, Thomas Benzing, Thomas Weimbs, Bernhard Schermer, Max C. Liebau

**Affiliations:** ^1^ Department of Pediatrics University of Cologne Faculty of Medicine and University Hospital Cologne Cologne Germany; ^2^ Department II of Internal Medicine University of Cologne Faculty of Medicine and University Hospital Cologne Cologne Germany; ^3^ Center for Molecular Medicine University of Cologne Faculty of Medicine and University Hospital Cologne Cologne Germany; ^4^ Institute of Pathology Faculty of Medicine University Hospital Cologne and University of Cologne Cologne Germany; ^5^ Nephrology and Dialysis Unit Bambino Gesù Children's Hospital IRCCS Rome Italy; ^6^ Pediatric Nephrology, Dialysis and Transplant Unit Fondazione IRCCS Cà Granda Ospedale Maggiore Policlinico Milan Italy; ^7^ CECAD University of Cologne Faculty of Medicine and University Hospital Cologne Cologne Germany; ^8^ Systems Biology of Ageing Cologne University of Cologne Cologne Germany; ^9^ Molecular, Cellular, and Developmental Biology, and Neuroscience Research Institute University of California Santa Barbara CA USA

**Keywords:** cilia, genetic kidney diseases, polycystic kidney disease

## Abstract

Autosomal recessive polycystic kidney disease (ARPKD) is mainly caused by variants in the *PKHD1* gene, encoding fibrocystin (FC), a large transmembrane protein of incompletely understood cellular function. Here, we show that a C‐terminal fragment of human FC can suppress a signalling module of the kinase SRC and signal transducer and activator of transcription 3 (STAT3). Consistently, we identified truncating genetic variants specifically affecting the cytoplasmic tail in ARPKD patients, found SRC and the cytoplasmic tail of fibrocystin in a joint dynamic protein complex and observed increased activation of both SRC and STAT3 in cyst‐lining renal epithelial cells of ARPKD patients.

## 1 INTRODUCTION

Autosomal recessive polycystic kidney disease (ARPKD) is a severe and early‐onset hepatorenal fibrocystic disease, mainly caused by variants in the *PKHD1* gene.^1^ *PKHD1* encodes a 450 kDa protein of poorly understood function termed fibrocystin (FC), which consists of a large extracellular part, a single transmembrane domain, and a short C‐terminal cytoplasmic tail. Post‐translational proteolytic cleavage and nuclear translocation of a C—terminal FC‐fragment have been described,^2^, ^3^ but an orthologous mouse model has recently questioned the functional relevance of the cytoplasmic tail.^4^

ARPKD and the more common autosomal dominant polycystic kidney disease (ADPKD) show overlapping clinical and genetic characteristics. Increased activation of the pro‐proliferative transcription factor STAT3 has previously been observed in preclinical PKD models and ADPKD patient samples.^5^ Using patient samples, we here describe activation of the SRC‐STAT3 axis in ARPKD cyst‐lining renal epithelia and furthermore show that a carboxy‐terminal fragment of human FC can control a SRC‐STAT3‐signalling module in cellular studies.

## 2 MATERIAL AND METHODS

### 2.1 Immunohistochemistry, co‐immunoprecipitation and western blot

For immunohistochemical staining, formalin‐fixed, paraffin‐embedded patient kidney tissue was used. Immunohistochemistry, co‐Immunoprecipitation and Western blot analyses were performed using standard methods. For details and antibodies, please see supplemental methods.

### 2.2 Stat3 luciferase reporter assay

HEK293T cells were seeded in a 96‐well plate. Plasmids were transfected using Lipofectamine 2000 (Invitrogen) and treated with 1 µM Forskolin as indicated. After cell lysis, firefly luciferase activity was detected using the Dual‐Luciferase® Reporter assay system (Promega) and the EnSpire® plate reader (Perkin Elmer).

### 2.3 Statistics

Data are expressed as datapoints of single n's with mean ± SEM of *n* experiments. Statistical evaluation was performed by using 2‐tailed Student's *t* test or repeated measures ANOVA with Tukey's post‐hoc analyses. *P* values less than 0.05 were considered significant.

## 3 RESULTS

**Figure 1:**
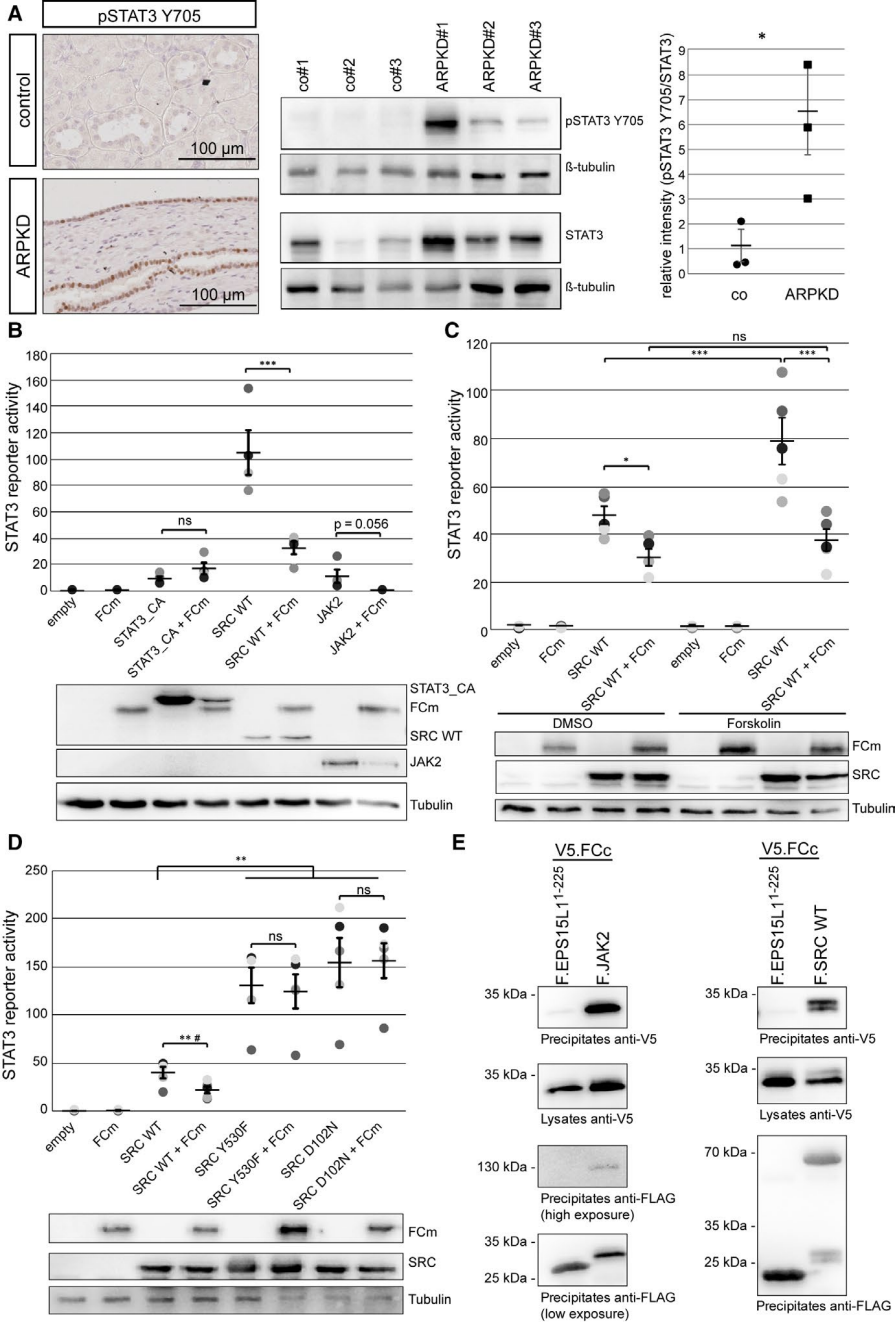
A C‐terminal fragment of human fibrocystin can inhibit STAT3‐activation by SRC. (A) Immunohistochemical staining of renal ARPKD and control tissue show increased STAT3 phosphorylation at tyrosine 705 (brown) in ARPKD cyst‐lining epithelia. Nuclei are counterstained in blue. Kidney lysates of three independent ARPKD patients and controls were stained for pSTAT3 Y705 and STAT3, and signal intensity was quantified. ARPKD patients show significantly increased STAT3 phosphorylation (t(4) = 2,890; *P* = .0223; *P < .05). (B‐C) STAT3 luciferase reporter assay showing that co‐expression of FCm reduced the activation of STAT3‐dependent transcription by SRC or JAK2 (N = 4; Repeated Measures ANOVA analysis demonstrated significant difference in STAT3 activation between groups (F(7,21) = 28.99, *P* < .0001; Tukey's post‐hoc ***P < .001)) (B). The effect was seen even after increase of intracellular cAMP concentrations by forskolin (N = 5; Repeated Measures ANOVA analysis demonstrated significant differences in STAT3 activation between groups (F(7,28) = 60.15, *P* < .0001; Tukey‘s post‐hoc *P < .05, **P < .001)) (C). There was no effect of FCm on a dimerizing STAT3 mutant (STAT3_CA) (B). (D) Constitutive active SRC variants (Y530F and D102N) activate STAT3 stronger than SRC WT. This activation cannot be reduced by FCm (N = 5; Repeated Measures ANOVA analysis demonstrated significant differences in STAT3 activation between groups (F(7,28) = 45.51, *P* < .0001)). #*T* test comparing the groups SRC WT with SRC WT + FCm only confirms significant difference in SRC‐induced STAT3 activation (t(4) = 5.779, *P* = .0022). (E) After exogenous expression FCc co‐precipitates with JAK2 and SRC, but not with a control protein. Co‐precipitating FCc appears as double band in the presence of SRC. In luciferase assays, different shades of grey represent independent experiments

In order to identify proteins interacting with the cytoplasmic tail of FC, we performed a yeast‐two hybrid screen and independent immunoprecipitation experiments in human embryonic kidney cells (Figure S1A/B) and identified STAT3 as a part of the FC protein complex. To examine pathophysiological aspects of STAT3 signalling in human ARPKD, we studied patient samples and found increased activation of STAT3 in human ARPKD cyst‐lining epithelia and both an increased expression and variably increased activation of STAT3 in human ARPKD kidney lysates (Figure 1A, Figure S1C, Suppl. methods for clinical and genetic information).

The relevance of functional studies on the cytoplasmic domain of human FC was supported by the identification of two patients with one truncating *PKHD1* variant affecting the cytoplasmic tail (patient A: frameshift variant c.11773_11774ins19 (p.Val3925fs) with c.5353T>C (p.Phe1785Leu); patient B: frameshift variant c.11901delG (p.Pro3968Leufs) with c.4292G>A (p.Cys1431Tyr)). Both patients show signs of portal hypertension, enlarged cystic kidneys and chronic kidney disease stage G4 in adolescence.

To study mechanisms of STAT3‐activation in ARPKD, we therefore used a C‐terminal fragment of human FC that contains a short extracellular part, the transmembrane domain and the cytoplasmic tail (FCm; Figure S1D) to allow posttranslational cleavage of FC.^2^, ^3^ The expression of FCm neither affected STAT3‐dependent transcription per se nor a constitutively active, dimerizing mutant of STAT3 (STAT3_CA; Figure 1B) in reporter assays. Next, we tested effects after co‐expressing the kinases JAK2 and SRC, two activators of STAT3 through different pathways. Co‐expression of FCm resulted in significantly diminished SRC‐induced STAT3‐activation with minor effects on JAK2‐induced STAT3‐activation (Figure 1B). Interestingly, the isolated cytoplasmic tail of FC (FCc) showed a similar, although less pronounced, effect on SRC‐induced STAT3‐activation (Figure S1D,E).

We then focussed on SRC‐STAT3‐signalling. An increase in intracellular cAMP concentration can enhance SRC activity and is considered to be a major driver in PKD.^1^ FCm could inhibit SRC‐STAT3‐activation by forskolin, a pharmacological activator of adenylate cyclase increasing cAMP levels (Figure 1C). The effect was lost for two constitutively active SRC variants (SRC^Y530F^ and SRC^D102N^) suggesting a direct FCm effect on SRC‐activation (Figure 1D). FCc could be found in joint protein complexes with SRC and JAK2 (Figure 1E). DZIP1L, a recently described *bona fide* ARPKD protein,^1^ was also detected in protein complexes with SRC and STAT3, but without effects on SRC‐STAT3‐dependent transcription (Figure S2).

**Figure 2:**
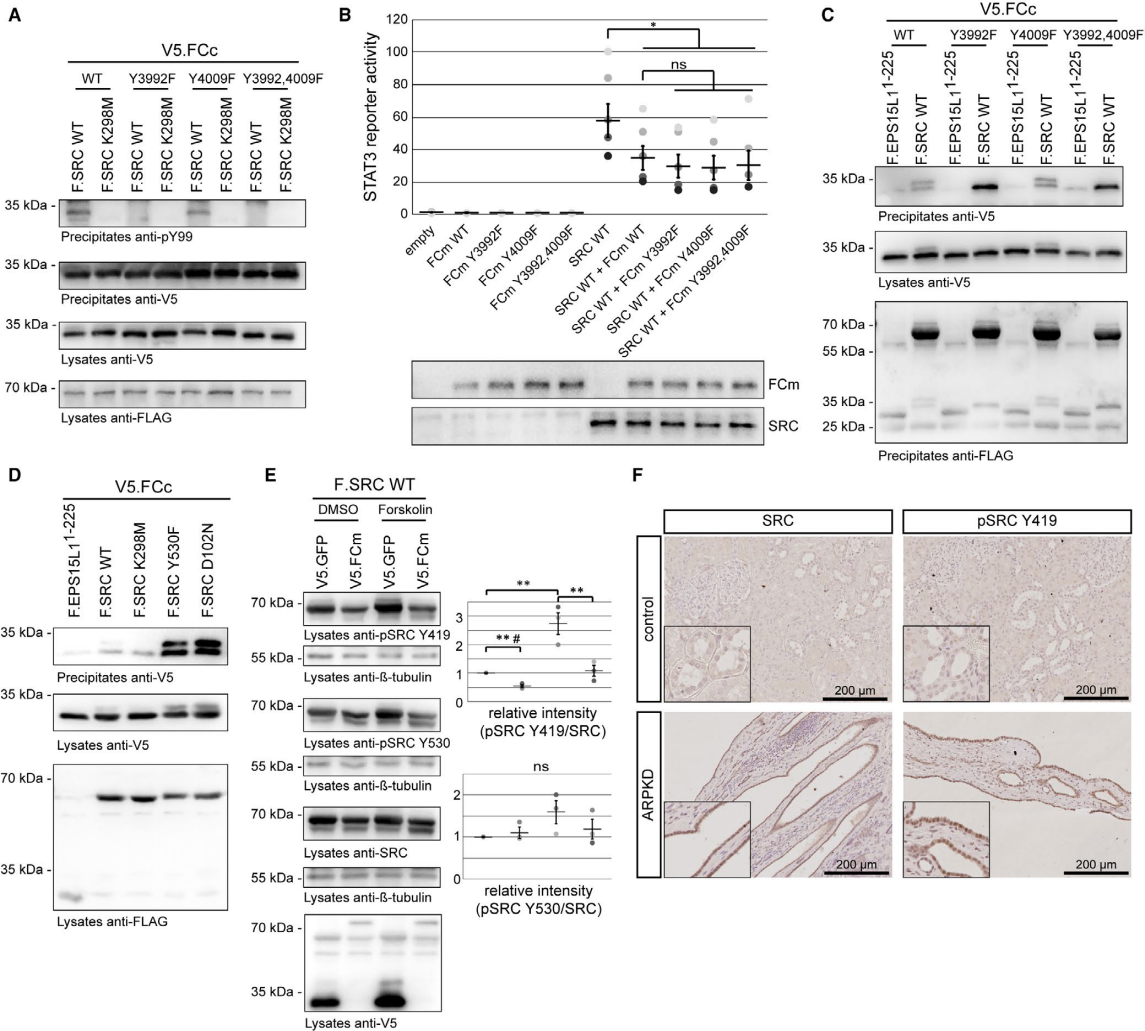
SRC induces tyrosine phosphorylation of FC at tyrosine 3992, shows dynamic co‐precipitation with FCc and is activated in ARPKD cyst‐lining epithelia. (A) HEK293T cells were transiently transfected with plasmids indicated. Tyrosine mutants of FCc were generated by site‐directed mutagenesis and precipitated in the presence of either SRC WT or the kinase dead SRC K298M mutant. SRC‐dependent tyrosine phosphorylation (pY99) of FCc is detected only for FCc WT and the FCc^Y4009F^ mutant, but not for the FCc^Y3992F^ or FCc^Y3992,4009F^ mutants. (B) STAT3 luciferase reporter assay did not show any functional effect of the tyrosine mutations in FCc (N = 5; Repeated Measures ANOVA analysis demonstrated significant differences in STAT3 activation between groups (F(9,36) = 20.63, *P* < .0001; Tukey‘s post‐hoc *P < .05)). (C) In co‐immunoprecipitation experiments, all four FCc variants (WT, Y3992F, Y4009F, Y3992,4009F) co‐precipitated with SRC. Double bands of co‐precipitating WT and FCc^Y4009F^ support tyrosine phosphorylation of FC’s tyrosine 3992 by SRC (D) *Vice versa*, FCc co‐precipitated with different variants of SRC (WT, K298M, Y530F, D102N). Co‐precipitation of FCc was stronger with constitutively active variants Y530F and D102N, and no double band was seen in the presence of the kinase‐dead variant K298M. (E) FCm or a control was co‐expressed with SRC in the presence or absence of forskolin. FCm significantly decreased the SRC phosphorylation on tyrosine 419 independent of forskolin. Phosphorylation on tyrosine 530 of SRC was unaffected (N = 3; Repeated Measures ANOVA analysis demonstrated significant differences in SRC activation between groups (F(3,6) = 18.20, *P* = .0020). #*T* test comparing these groups only shows significant difference in SRC phosphorylation at tyrosine 419 (t(2) = 8.702, *P* = .0065). (F) Immunohistochemistry staining shows increased expression and activation of SRC in ARPKD kidney tissue. In luciferase assays, different shades of grey represent different independent experiments

SRC, but not JAK2, induced a double band of human FCc (Figure 1E). To study potential tyrosine phosphorylation, we mutated both tyrosine residues in FCc (Y3992, Y4009) to phenylalanine. Mutation of tyrosine 3992 alone (Y3992F) and of both tyrosines (Y3992,4009F), but not of tyrosine 4009 alone (Y4009F), led to a loss of the SRC‐induced phosphotyrosine signal of precipitated FCc, suggesting SRC‐induced phosphorylation at the non‐conserved tyrosine 3992 (Figure 2A). Mutation of the tyrosines had no functional effects on the regulation of the SRC‐STAT3‐axis (Figure 2B) or FC‐SRC‐co‐precipitation. For WT and the Y4009F mutant, a double band of co‐precipitated FCc was seen, supporting SRC‐induced tyrosine phosphorylation at tyrosine 3992 (Figure 2C). Interestingly, FCc co‐precipitation was more pronounced with activated SRC variants (Figure 2D). We therefore speculated that FCm might induce its effect on STAT3‐dependent transcription by inhibiting SRC‐activation. Indeed, expression of FCm resulted in a significantly reduced signal for the activating phosphorylation of SRC at tyrosine 419 with and without forskolin (Figure 2E). Strikingly, this phosphorylation was strongly increased in cyst‐lining epithelia of ARPKD kidneys (Figure 2F).

## 4 DISCUSSION

The pathophysiology of cystogenesis in ARPKD and the molecular function of fibrocystin are not well understood. Overlapping functions with the ADPKD proteins have been suggested.^6^, ^7^, ^8^ We show that the C‐term of human FC can regulate SRC‐activation and that SRC and STAT3‐activation can be observed in ARPKD cyst‐lining epithelial cells. Interestingly, pharmacological inhibition of SRC‐activation results in amelioration of the phenotypes in various preclinical models of PKD.^9^, ^10^, ^11^ First clinical trials on SRC inhibitors for PKD have been initiated (eg ClinicalTrials.gov identifiers NCT03096080 and NCT03203642).^12^ SRC can be activated through multiple mechanisms, including increased intracellular cAMP concentration,^5^ but details in PKD remain elusive.

We found SRC and FCc in a common protein complex fitting to previous data linking FC to focal adhesion complexes.^13^ Our data suggest that FCm can control the activation of SRC and may thus have a partly opposing function to the polycystin‐1 C‐term that activates SRC‐induced STAT3‐dependent transcription.^5^

Our data have two important implications: Firstly, the pathogenic relevance of fibrocystin's cytoplasmic tail has been questioned as deletion of the murine FC C‐term does not result in the liver phenotype observed in other *Pkhd1*‐deficient models.^4^ Yet, the cytoplasmic tail is poorly conserved and *Pkhd1*‐deficient mice do not develop a renal phenotype resembling human ARPKD.^6^, ^8^, ^14^ Our genetic findings and the experimental cell culture data support the hypothesis that FC’s cytoplasmic tail may indeed be functionally important in humans. The transmembrane domain and post‐translational processing may be required for full functionality. Secondly, the data suggest activation of SRC‐STAT3‐signalling in cyst‐lining renal epithelia in ARPKD bearing therapeutic potential. This activation showed some variability amongst patients with different clinical courses. Larger numbers and additional studies on the interplay between cyst‐lining epithelial signalling and, for example, interstitial inflammatory processes, functional mapping in full‐length fibrocystin, and studies on the effects of the observed SRC‐induced FC phosphorylation on the non‐conserved tyrosine 3992 are required.^15^

In summary, we show increased activation of SRC and STAT3 in cyst‐lining epithelia of ARPKD kidneys. Mechanistically, our data suggest that the carboxy‐terminus of human FC can contribute to control of SRC‐activation.

## CONFLICT OF INTEREST

Dr Weimbs has a patent Novel Treatment for Polycystic Kidney Disease with royalties paid by Chinook Therapeutics, and a patent Methods and Compositions for Supporting Renal Health with royalties paid by Santa Barbara Nutrients, Inc and Consultant and sponsored research, Chinook Therapeutics Founder, shareholder and managerial position at Santa Barbara Nutrients, Inc. Dr Liebau reports advisory board activities for Otsuka Pharmaceuticals as a representative of the University Hospital Cologne, and personal fees from Pfizer, outside the submitted work. The other authors do not have anything to disclose.

## AUTHOR CONTRIBUTIONS

**Claudia Dafinger:** Conceptualization (equal); Formal analysis (lead); Investigation (lead); Methodology (equal); Visualization (lead); Writing‐original draft (equal). **Amrei M. Mandel:** Conceptualization (equal); Formal analysis (equal); Funding acquisition (supporting); Investigation (lead); Visualization (equal); Writing‐original draft (equal). **Alina Braun:** Formal analysis (supporting); Funding acquisition (supporting); Investigation (equal); Writing‐review & editing (equal). **Heike Goebel:** Formal analysis (supporting); Investigation (supporting); Resources (supporting); Visualization (equal); Writing‐review & editing (equal). **Kathrin Burgmaier:** Data curation (equal); Funding acquisition (supporting); Investigation (supporting); Resources (equal); Writing‐review & editing (equal). **Laura Massella:** Investigation (supporting); Resources (supporting); Writing‐review & editing (equal). **Antonio Mastrangelo:** Investigation (supporting); Resources (supporting); Writing‐review & editing (equal). **Jörg Dötsch:** Funding acquisition (supporting); Project administration (supporting); Resources (equal); Supervision (supporting); Writing‐review & editing (equal). **Thomas Benzing:** Conceptualization (equal); Funding acquisition (equal); Project administration (supporting); Resources (equal); Supervision (equal); Writing‐review & editing (equal). **Thomas Weimbs:** Conceptualization (equal); Funding acquisition (supporting); Methodology (equal); Project administration (supporting); Resources (equal); Supervision (supporting); Validation (supporting); Writing‐review & editing (equal). **Bernhard Schermer:** Conceptualization (equal); Formal analysis (supporting); Funding acquisition (equal); Investigation (supporting); Methodology (supporting); Project administration (supporting); Resources (equal); Supervision (equal); Validation (equal); Writing‐original draft (supporting); Writing‐review & editing (equal). **Max Christoph Liebau:** Conceptualization (lead); Data curation (supporting); Formal analysis (supporting); Funding acquisition (lead); Investigation (supporting); Project administration (lead); Resources (equal); Supervision (lead); Validation (lead); Visualization (supporting); Writing‐original draft (lead); Writing‐review & editing (lead).

## Supporting information

Supplementary Material

## Data Availability

The data that support the findings of this study are available from the corresponding author upon reasonable request.
