# Supplementary material for: The carboxy‐terminus of the human ARPKD protein fibrocystin can control STAT3 signalling by regulating SRC‐activation
Source: J Cell Mol Med. 2020 Oct 28;24(24):14633–8. doi: 10.1111/jcmm.16014 (PMC7754027; doi:10.1111/jcmm.16014)
Supplement: Supplementary file 1 — Supplementary Material [file JCMM-24-14633-s001.docx]

**Suppl. information to the manuscript**

**The carboxy-terminus of the human ARPKD protein fibrocystin can control STAT3 signalling by regulating SRC activation**

Claudia Dafinger, Amrei M. Mandel, Alina Braun, Heike Göbel, Kathrin Burgmaier, Laura Massella, Antonio Mastrangelo, Jörg Dötsch, Thomas Benzing, Thomas Weimbs, Bernhard Schermer, Max C. Liebau

**Suppl. Figure 1. FCc and STAT3 can be found in a common protein complex and FCc negatively regulates SRC-induced STAT3-dependent transcription.** **(A-B)** STAT3 co-precipitates with the cytoplasmic tail of FC (FCc, FC^3882-4074^), but not with a control protein (A), and vice versa (B). **(C)** Immunohistochemistry stainings of different renal ARPKD and control tissues show increased Stat3 phosphorylation (Y705) in cyst epithelia of independent ARPKD samples. Nuclei are stained in blue. The upper right image of the ARPKD panel represents a broader overview of the sample presented in Figure 1A. The four pictures represent stainings of samples from four kidneys of three independent ARPKD patients, two of which with proven *PKHD1* variants (upper pictures). **(D)** Graphical presentation of the fibrocystin fragments used in this study. **(E)** FCc negatively regulates SRC-induced STAT3-dependent transcription in a similar but less pronounced way as FCm, (N=7; Repeated Measures ANOVA analysis demonstrated significant differences in STAT3 activation between SRC and SRC + FCc (F(3,18)=81.9, p<0.0001; Bonferroni's post-hoc *p<0.05)). In luciferase assays different shades of grey represent independent experiments.

**Suppl. Figure 2. DZIP1L can be found in protein complexes with STAT3 and SRC but does not influence SRC-induced STAT3-dependent transcription. (A-B)** STAT3 (A) and SRC (B) co-precipitate with DZIP1L, but not with a control protein. **(C)** DZIP1L does not influence STAT3 activity directly or via SRC in a STAT3 luciferase reporter assay (N=4; Repeated Measures ANOVA analysis demonstrated significant differences in STAT3 activation between groups (F(10,30)=15.75, p<0.0001; Tukey‘s post-hoc)). In luciferase assays different shades of grey represent independent experiments.


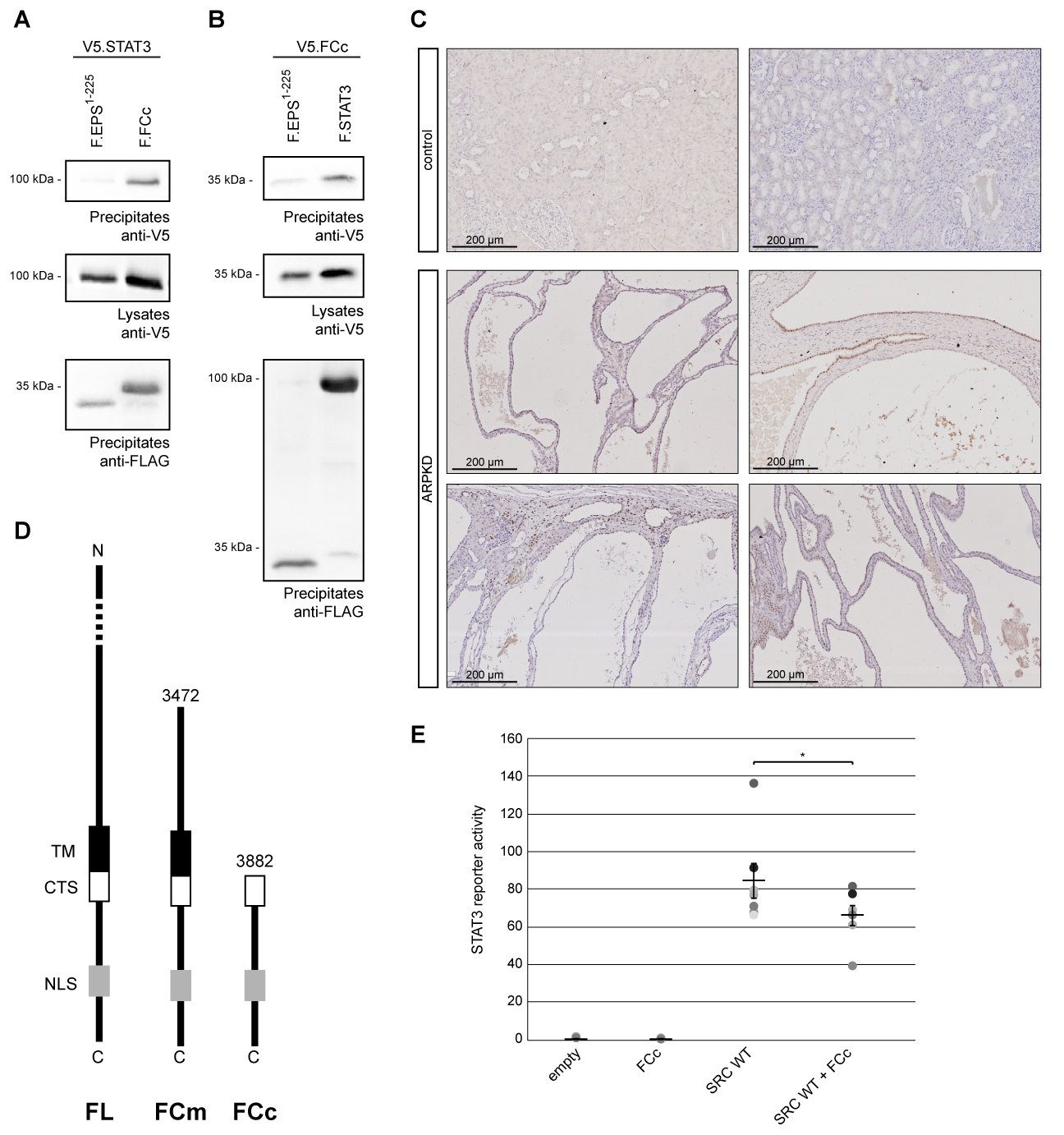


**Suppl. Figure 1**

**
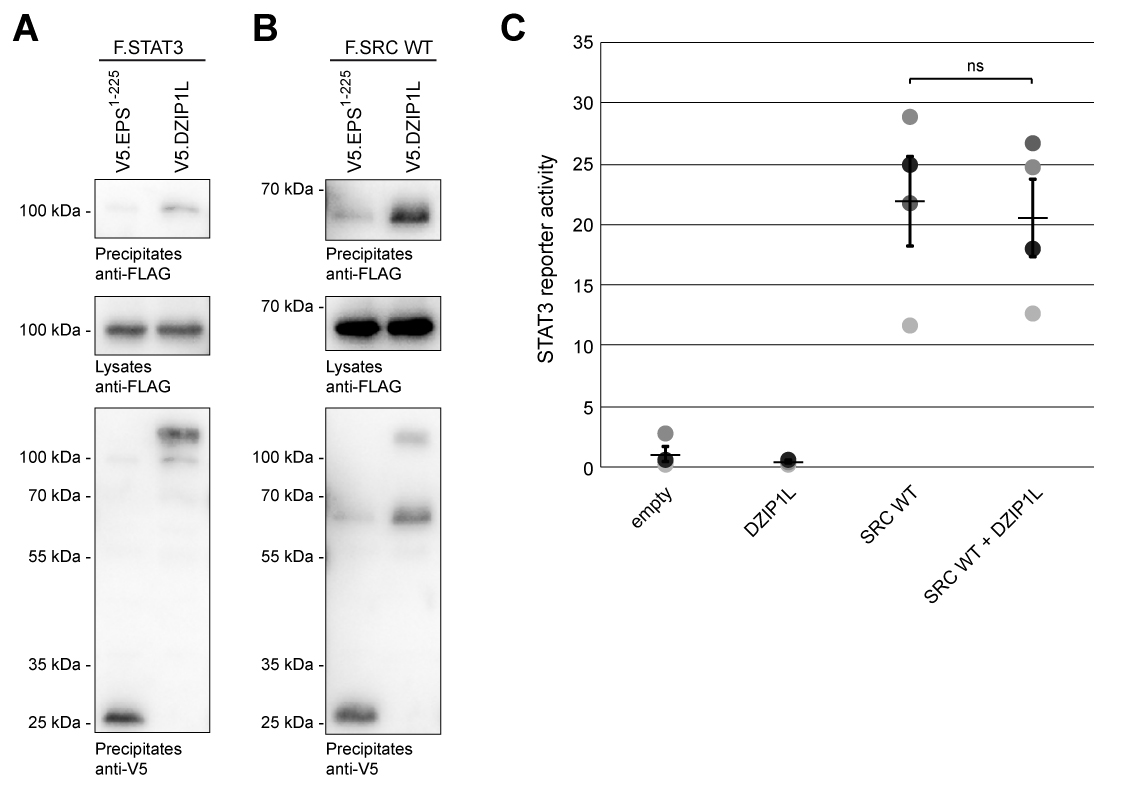
**

**Suppl. Figure 2**

**Supplemental methods.**

**Patient samples and cell lines.**

Samples of from ARPKD kidneys after nephrectomies from three independent patients were used for immunohistochemistry and for Western blot analysis of STAT3 expression and activation in ARPKD kidneys. Normal tissue from tumor nephrectomies served as control tissue. ARPKD kidneys were directly taken from operating theatre to pathology. Two of the three patients underwent genetic testing and showed *PKHD1* variants. One of the patients has previously been reported by our group (6). The genotype of patient 1 was c.8011C>T (p.Arg2671*); c.51A>G (p.Val18Glyfs*3), thus showing two pathogenic *PKHD1* variants resulting in extracellular truncation of fibrocystin (ACMG class 5). Patient 2 showed one ACMG class 4 *PKHD1* variant (likely pathogenic) c.9829+1 G>A; and two *PKHD1* variants of unknown significance (ACMG class 3) c.2990T>A (p.Met997Lys); c.334G>A (p.Gly112Arg). All patients showed a classical clinical ARPKD phenotype with massively enlarged kidneys Patient 1 underwent nephrectomy at the age of three years and had been without dialysis up to this point, while both patient 2 and patient 3 underwent nephrectomy within the first months of life and had a very early need for peritoneal dialysis. The use of patient samples occurred after approval by the Ethics Committee of the Faculty of Medicine of Cologne University. HEK293T cells were cultured in DMEM medium (Sigma-Aldrich) containing 10% FBS under standard conditions (37°C, 5% CO_2_).**Immunohistochemistry.**

Formalin-fixed, paraffin-embedded tissue was cut in 2 µm thin slides. Paraffin was removed by xylene treatment following rehydration in graded ethanol. Antigen retrieval was achieved by 10 min boiling in 10 mM citric acid buffer. Endogenous peroxidase reactions and unspecific antibody binding sites were blocked by treatment with 3% H2O2 and Avidin/Biotin blocking (Vector Laboratories, SP-2001) according to the manufacturer’s protocol or incubation with 5% bovine serum albumine, respectively. After incubation with primary and secondary antibodies the slides were labelled with Vectastain R.T.U Elite ABC Reagent (Vector Laboratories, PK-7100) and developed with Pierce^TM^ DAB Substrate Kit (Thermo Scientific, 34002). For nuclear counter staining the slides were treated with Mayer’s Haemalaun (Sigma-Aldrich). Slides were finally dehydrated in raising ethanol and mounted with Histomount.

**Plasmids.**

Truncations of the coding sequences of human *PKHD1* (3882-4074, 3472-4074), *SRC* WT, *JAK2, DZIP1L* and murine Stat3 were cloned by standard PCR amplification techniques from human kidney cDNA library or HEK293T cDNA or murine cDNA, respectively. The *GFP* and *EPS15L1* plasmids have previously been described (1–4). Point mutations were introduced by quick change PCR. All sequences were confirmed. The Stat1/3 luciferase reporter plasmid was a kind gift of Thomas Weimbs. The Renilla luciferase plasmid pGL4.74 was obtained from Promega (E6921). Fibrocystin (Y3992F; Y4009F; Y3992,4009F) and SRC (Y530F; D102N; K298M=KD) mutants were generated by site-directed mutagenesis PCR. The Stat3 mutant has previously been described (5).

**Antibodies.**

Antibodies were obtained from Cell Signaling (anti-SRC #2110), Thermo Fisher (anti-pSRC Y419 #44-660G, anti-pSRC Y530 #44-662G), Santa Cruz (anti-pY99 #sc-7020), Serotec (anti-V5 #MCA1360), Millipore (anti-V5 #AB3792), Sigma-Aldrich (anti-FLAG #F7425), and DSHB (anti-ß-tubulin #E7).

**Co-Immunoprecipitation.**

HEK293T cells were transfected with plasmid DNA (as indicated) using a modified calcium phosphate method. After 24 h proteins were isolated by lysing the cells in lysis buffer (1% Triton X-100, 20 mM Tris pH 7.5, 25 mM NaCl, 50 mM NaF, 15 mM Na_4_P_2_O_7_, 1 mM EDTA, 0.25 mM PMSF, and 5 mM Na_3_VO_4_) on ice for 15 min and centrifuging (20,000 g, 4°C, 15 min). Flag-tagged proteins were pulled out of the supernatant using anti-Flag antibody-coupled sepharose beads (M2-beads, Sigma-Aldrich); V5-tagged proteins were precipitated using an anti-V5 antibody and Protein G beads. After a binding time of 1-24 h the beads were washed thrice with lysis buffer and boiled in 2xLaemmli containing 0.1 M DTT for protein denaturation.

**Western blot.**

Protein samples were separated by 10% SDS-PAGE (Invitrogen system) and blotted on a PVDF membrane (Millipore). The membrane was blocked for 30 min in 5% BSA (PAA) and incubated with the indicated primary antibodies over night at 4°C. After incubation with the appropriate horseradish peroxidase-coupled secondary antibodies (Dako), the proteins were visualized using homemade enhanced chemiluminescence technology (100 mM Tris, 1.25 mM luminol, 0.2 mM coumaric acid, 0.75% H_2_O_2_, pH 8.5).

**References**

1. Benzing T, Gerke P, Höpker K, Hildebrandt F, Kim E, Walz G. Nephrocystin interacts with Pyk2, p130(Cas), and tensin and triggers phosphorylation of Pyk2. Proc Natl Acad Sci USA. 14. August 2001;98(17):9784–9.

2. Otto EA, Schermer B, Obara T, O’Toole JF, Hiller KS, Mueller AM, u. a. Mutations in INVS encoding inversin cause nephronophthisis type 2, linking renal cystic disease to the function of primary cilia and left-right axis determination. Nat Genet. August 2003;34(4):413–20.

3. Loges NT, Olbrich H, Becker-Heck A, Häffner K, Heer A, Reinhard C, u. a. Deletions and point mutations of LRRC50 cause primary ciliary dyskinesia due to dynein arm defects. Am J Hum Genet. Dezember 2009;85(6):883–9.

4. Borgal L, Habbig S, Hatzold J, Liebau MC, Dafinger C, Sacarea I, u. a. The ciliary protein nephrocystin-4 translocates the canonical Wnt regulator Jade-1 to the nucleus to negatively regulate β-catenin signaling. J Biol Chem. 20. Juli 2012;287(30):25370–80.

5. Liddle FJ, Alvarez JV, Poli V, Frank DA. Tyrosine phosphorylation is required for functional activation of disulfide-containing constitutively active STAT mutants. Biochemistry. 2. Mai 2006;45(17):5599–605.

6. Ebner K, Dafinger C, Ortiz-Bruechle N, Koerber F, Schermer B, Benzing T, u. a. Challenges in establishing genotype-phenotype correlations in ARPKD: case report on a toddler with two severe PKHD1 mutations. Pediatr Nephrol. Juli 2017;32(7):1269–73.
